# Physics-Guided Deep Learning for Interpretable Biomedical Image Reconstruction and Pattern Recognition in Diagnostic Frameworks

**DOI:** 10.3390/bioengineering13040457

**Published:** 2026-04-13

**Authors:** Akeel Qadir, Saad Arif, Prajoona Valsalan, Osama Khan

**Affiliations:** 1 School of Information Engineering, Xi’an Eurasia University, Xi’an 710065, China; 2 Research Center of Smart Sensing Chips, Ningbo Institute of Northwestern Polytechnical University, Ningbo 315103, China; 3 Department of Mechanical Engineering, College of Engineering, King Faisal University, Al-Ahsa 31982, Saudi Arabia; osamakhan6165@gmail.com; 4 Department of Electrical and Computer Engineering, Dhofar University, Salalah 211, Oman; pvalsalan@du.edu.om

**Keywords:** AI-assisted diagnostic workflows, automated disease classification, biomedical image reconstruction, clinical decision support systems, deep learning-based image segmentation, explainable artificial intelligence, physics-informed deep learning, predictive imaging biomarkers, radiomics-based feature extraction, translational medical imaging

## Abstract

This study introduces a physics-guided deep learning architecture designed for the simulation, reconstruction, and pattern recognition of biomedical images. By explicitly integrating physical priors into the learning model, the framework addresses the black-box nature of traditional artificial intelligence (AI). It provides an explainable AI pathway that enhances diagnostic accuracy, robustness, and clinical interpretation. The proposed framework was evaluated through systematic simulation studies. It involved complex geometric configurations, multimodal physical fields, and noise-corrupted synthetic three-dimensional brain volumes. Quantitative analysis demonstrates consistent improvements in reconstruction fidelity, with the peak signal-to-noise ratio (PSNR) reaching 47 dB and the structural similarity index exceeding 0.90 across all scenarios. Notably, at moderate noise levels (0.05), the framework maintains a PSNR greater than 32 dB, ensuring structural integrity essential for computer-aided diagnosis. Volumetric brain experiments further reveal a 38–44% reduction in activation localization errors, highlighting the framework’s utility in functional imaging and disease prognosis. By grounding deep learning in physical constraints, this study provides a transparent and robust solution for automated disease classification and advanced biomedical imaging tasks within clinical decision support systems.

## 1. Introduction

Biomedical image simulation, reconstruction, and pattern recognition are widely used in contemporary diagnostic and functional image modalities. It includes magnetic resonance imaging (MRI), computed tomography (CT), positron emission tomography (PET), and functional brain imaging [1,2,3], etc. The increasing demand for high resolution, robustness to noise, and data efficiency in imaging has led to the adoption of deep learning (DL) methods. However, purely data-driven models tend to exhibit limited generalizability, noise sensitivity, and poor physical interpretability. These limitations are particularly critical in biomedical settings; where data quality may be limited, data acquisition conditions may vary significantly, and high model accuracy is required for clinical decision making. To address these challenges, physics-informed and physics-guided DL approaches have emerged as viable solutions by incorporating established physical laws, geometric constraints, and field dynamics into learning systems [4]. Such approaches enable the generation of realistic training data through physics-based simulations with DL. They enforce physically consistent solutions and enhance robustness to noise and structural variability [5]. The objective of this study is to develop a physics-inspired DL model for biomedical image simulation, reconstruction, and pattern recognition. It can simulate interactions of complex 2D and 3D fields, geometric inclusions, and volumetric activation patterns [6]. The proposed framework is intended to provide a flexible and scalable simulation platform for the development and evaluation of DL models in biomedical imaging. Therefore, it bridges the gap between physics-based modeling and data-driven intelligence [7,8,9].

Recent developments in biomedical imaging have employed DL methods for image reconstruction, segmentation, and pattern recognition. However, purely data-driven models often fail to maintain structural fidelity, generalize across different anatomical geometries, or remain stable under noisy or low-quality imaging conditions. A physics-informed neural network (PINN) framework was introduced for myocardial perfusion MRI to estimate tracer-kinetic parameters by embedding multi-compartment exchange model constraints [10]. Rank-based selective ensemble DL methods were proposed for invasive coronary angiography segmentation [11]. A model-driven PINN for magnetic resonance electrical property tomography was proposed [12]. It enabled artifact-reduced reconstruction of tissue electrical properties by embedding Maxwell-based physical constraints directly into the learning process. A PINN-based biomechanical analysis framework was developed to achieve globally optimal stress estimation. It also achieved automatic bone composition differentiation from small-sample spinal CT data by incorporating feature-aware weight initialization and multi-physics constraints [13].

To address limitations in real-time 3D tissue imaging, a hybrid PINN was proposed. It integrated recurrent neural networks with a differentiable fluid solver to reconstruct flow-induced 3D tissue dynamics from sparse 2D observations [14]. In cerebrovascular assessment, a physics-informed DL framework was developed that fused sparse transcranial Doppler measurements with reduced-order hemodynamic simulations. It reconstructed high-resolution, physically consistent maps of cerebral blood velocity, pressure, and vessel area across the brain vasculature [15]. To address the clinical complexity of conventional hemodynamic analysis, another study developed an input-parameterized PINN (IP-PINN). It enabled fast and accurate hemodynamic parameter estimation without requiring specialized expertise [16]. Building on solid mechanics principles, a study proposed an unsupervised physics-aware machine learning framework for medical image registration. It coupled large-deformation elasticity with growth and remodeling biophysics. It demonstrated accurate alignment under complex deformations and competitive performance across brain and developmental imaging benchmarks [17].

To position this work, a distinguishment is made between physics-informed and physics-guided learning. Conventional PINN-based approaches primarily enforce physical laws through loss function constraints during training [18,19,20]. In contrast, the proposed framework adopts a physics-guided strategy across the entire pipeline by incorporating physical priors in spatial modeling, structural design, data generation, and learning. Unlike standard PINNs, the proposed method introduces a multistage, simulation-driven framework for biomedical image reconstruction and pattern recognition. Its novelty lies in the integration of physics at multiple stages and the ability to enable robust learning under varying geometry, modality, and noise conditions. Therefore, it extends beyond conventional PINN formulations [21,22].

Despite significant progress in physics-informed and physics-guided DL for biomedical imaging, several limitations remain in the literature [23,24]. Many existing approaches rely on large volumes of high-quality data, limiting their applicability to small datasets or rare diseases [25]. Furthermore, most methods are modality-specific and task-oriented, reducing their generalizability across different imaging domains [26]. Additional challenges include sensitivity to noise measurement and high computational requirements for volumetric (3D) data [27]. Simplification of assumptions is also needed in underlying physical models that may not fully capture complex anatomical or functional dynamics. These limitations highlight the need for a unified, robust, and physically consistent framework. It should be capable of accurate simulation, reconstruction, and pattern recognition across diverse biomedical imaging scenarios. As summarized in Table 1, existing physics-informed and physics-guided DL methods exhibit these limitations.

The primary contributions of this work are summarized as follows. A physics-guided DL (PGDL) architecture is presented in which physical priors are incorporated into biomedical image simulation, reconstruction, and pattern recognition. It analyses the problem under varying geometric, modality, noise, and volumetric conditions through large-scale simulations. Significant improvements in reconstruction accuracy, structural fidelity, and robustness are demonstrated. The key contributions are as follows:

1. A unified PGDL model is proposed for biomedical image simulation, reconstruction, and pattern recognition. Physical priors are explicitly incorporated in it to enhance accuracy, robustness, and interpretability.
2. Comprehensive multi-scenario validation is conducted through controlled simulation studies. It involved varying numbers of inclusions, complex geometric structures, multimodal physical fields, noise-corrupted measurements, and synthetic three-dimensional (3D) brain volumes. These aspects represented realistic biomedical imaging conditions.
3. Consistent quantitative performance improvements are achieved across all scenarios. It included 32–45% reduction in root mean square error (RMSE) and 4–7 dB increment in peak signal-to-noise ratio (PSNR). Structural similarity index measure (SSIM) exceeded 0.90 demonstrating superior reconstruction fidelity compared to unconstrained data-driven methods.
4. Under moderate noise conditions (σ = 0.05), strong noise robustness and structural preservation are maintained (PSNR > 32 dB and structural degradation < 5%). It indicated suitability for low signal-to-noise biomedical imaging environments.
5. Scalability to volumetric and functional imaging is demonstrated using synthetic 3D brain data. It demonstrated 38–44% reduction in localization error and consistent performance across scales which support applications in functional brain imaging and volumetric pattern recognition.

The remainder of this paper is organized as follows. Section 2 presents the theoretical background and mathematical modeling of the proposed PGDL framework. It includes physics-informed constraints and image reconstruction methodology. Section 3 describes the methodology, including simulation setup, data generation, and implementation details. Section 4 presents the results and discussion, including qualitative visualizations and quantitative performance evaluation. It reports RMSE, PSNR, and SSIM, and robustness analyses across various biomedical imaging scenarios. Section 5 concludes the paper by summarizing the key findings, contributions, and potential applications of the proposed framework in biomedical imaging.

## 2. Mathematical Modeling of the Proposed Physics-Guided Deep Learning Framework

This section presents the mathematical foundations of the proposed PGDL framework. The objective is to integrate physical constraints, geometrical priors, and noise modeling directly into the DL pipeline for biomedical image simulation, reconstruction, and pattern recognition. The modeling approach consists of field representation, geometric inclusion, volumetric activation modeling, noise incorporation, and physics-guided neural network formulation.

### 2.1. Physics-Based Field Representation

Let $\Omega \subset R^{d}$ (d = 2 or 3) denote the spatial domain of interest. The biomedical image intensity is modeled as a continuous physical field u(x) over this domain. To capture the physical nature of the imaging process, the field is expressed as a parametric function:

(1) u(x)=f(x;θ)

where f(⋅) denotes a physics-based field model and θ represents physical parameters such as diffusion coefficients, frequency components, or intensity decay rates. Examples of field types include:

- Gaussian fields for localized tissue activations;
- Wave fields for oscillatory imaging patterns;
- Stepwise intensity fields for modeling abrupt tissue transitions.

This formulation ensures that the generated image fields are physically consistent, smooth, and continuous, thereby providing a reliable foundation for network learning. In biomedical imaging, the image intensity can be associated with underlying physical processes. These include diffusion, wave propagation, or metabolic activity, etc., which are governed by biological and physical laws. Parametric field equations are used in the proposed framework to model these processes, thereby enabling the generation of physically meaningful image patterns. This approach ensures smoothness, locality, and structural consistency, which are often violated by unconstrained learning models [28]. Physics-grounded formulation provides a conceptual basis for simulating realistic imaging conditions and serves as a prior for DL-based reconstruction and recognition tasks [29]. In this context, PGDL integrates physical laws governing biomedical imaging processes into data-driven neural networks to improve reconstruction accuracy, robustness, and interpretability. Let

(2) u(x)∈R

denote a biomedical image field (e.g., intensity, activation, or concentration) defined over a spatial domain:

(3) $$\Omega \subset R^{d},\;d\in \{2,3\}$$

The physical behavior of the imaging field is generally governed by a partial differential equation:

(4) L(u(x))=f(x), x∈Ω

subject to boundary conditions:

(5) B(u(x))=g(x), x∈∂Ω

where L(⋅) is a differential operator (e.g., diffusion, wave, or transport operator), and f(x) represents internal sources or activations.

### 2.2. Geometric Inclusion and Boundary Modeling

Biological tissues exhibit heterogeneous structures with distinct spatial boundaries. Let $\Omega_{\mathrm{incl}}\subset \Omega$ denote the region of an inclusion (representing tissue heterogeneity, lesions, or anomalies). The field is modified within the inclusion as:

(6) $$u(x)=\left\{\begin{array}{cc} f(x;\theta ), & x\in \Omega_{\mathrm{incl}}\\ 0, & x\in \Omega \setminus \Omega_{\mathrm{incl}} \end{array}\right.$$

Multiple inclusions with varying shapes (circular, elliptical, rectangular) and sizes can be superimposed to simulate complex anatomical structures. This step introduces sharp boundaries and discontinuities, thereby enabling the network to learn robust reconstruction across heterogeneous tissues.

### 2.3. Volumetric Activation Modeling

For 3D biomedical imaging, a synthetic brain volume $\Omega_{B}$ is defined as:

(7) $$\Omega_{B}=\left\{(x,y,z)\in R^{3}|\frac{x^{2}}{a^{2}}+\frac{y^{2}}{b^{2}}+\frac{z^{2}}{c^{2}}\leq 1\right\}$$

Functional activations within this volume are modeled as localized Gaussian distributions:

(8) $$u_{B}(x,y,z)=exp(-\alpha ((x-x_{0}{)}^{2}+(y-y_{0}{)}^{2}+(z-z_{0}{)}^{2}))$$

where $\left(x_{0},y_{0},z_{0}\right)$ represents the activation center and α controls the spatial spread. This volumetric modeling enables realistic simulation of functional brain activity. Therefore, it provides representative 3D training and validation datasets for the DL model.

### 2.4. Noise Modeling

To simulate real-world biomedical imaging conditions, additive noise is incorporated into the field:

(9) $$\tilde{u}(x)=u(x)+\eta (x)$$

where η(x) represents stochastic noise, typically modeled as a Gaussian process with zero mean and variance $\sigma_{n}^{2}$. This formulation ensures that the DL network learns robust reconstruction under measurement uncertainty, enhancing generalization to real imaging scenarios.

### 2.5. Physics-Guided Deep Learning Formulation

Let $N_{\theta }(\cdot )$ denote a neural network with parameters θ that approximate the mapping from noisy measurements $\tilde{u}$ to clean, physically consistent image fields u. The network is trained by minimizing a physics-guided loss function:

(10) $$\begin{array}{c} L(\theta )=\lambda_{d}\underset{\mathrm{Data}\;\mathrm{fidelity}}{\underset{⏟}{\frac{1}{N}\sum_{i=1}^{N}∥N_{\theta }(x_{i})-u_{i}{∥}^{2}}}+\lambda_{p}\underset{\mathrm{Physics}\;\mathrm{residual}}{\underset{⏟}{\frac{1}{M}\sum_{j=1}^{M}∥R(N_{\theta }(x_{j})){∥}^{2}}}\\ +\lambda_{b}\underset{\mathrm{Boundary}\;\mathrm{constraint}}{\underset{⏟}{\frac{1}{K}\sum_{k=1}^{K}∥B(N_{\theta }(x_{k}))-g_{k}{∥}^{2}}}. \end{array}$$

where:

- R(⋅) enforces physical laws (e.g., smoothness, diffusion, or wave propagation constraints);
- B(⋅) enforces boundary conditions at domain edges or inclusions;
- $\lambda_{d},\lambda_{p},\lambda_{b}$ are weight coefficients that balance data fidelity and physical constraints;
- $g_{k}$ denotes prescribed (ground-truth) boundary condition values at sampled boundary points $x_{k}$.

**Figure 1:**
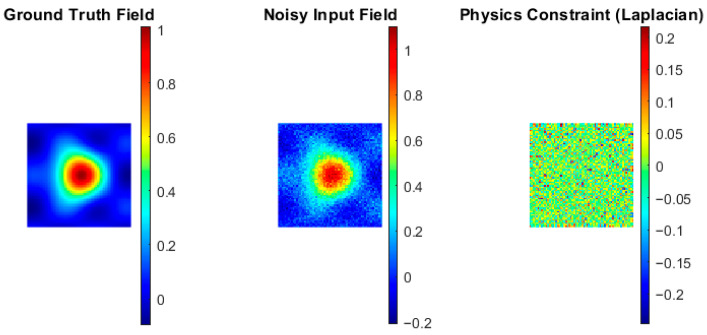
Conceptual physics-guided deep learning model including ground truth field, noisy input, and Laplacian-based physics constraint.

This formulation ensures that the network learns output that is consistent with both observed measurements and underlying physical principles. It results in improvement of accuracy, robustness, and interpretability. As shown in Figure 1, the proposed PGDL framework incorporates physical priors to guide network training using the ground truth field, noisy input, and Laplacian-based physics constraints. A concise algorithmic summary of the proposed PGDL framework is provided in Appendix A.

## 3. Methodology of the Proposed Framework

**Figure 2:**
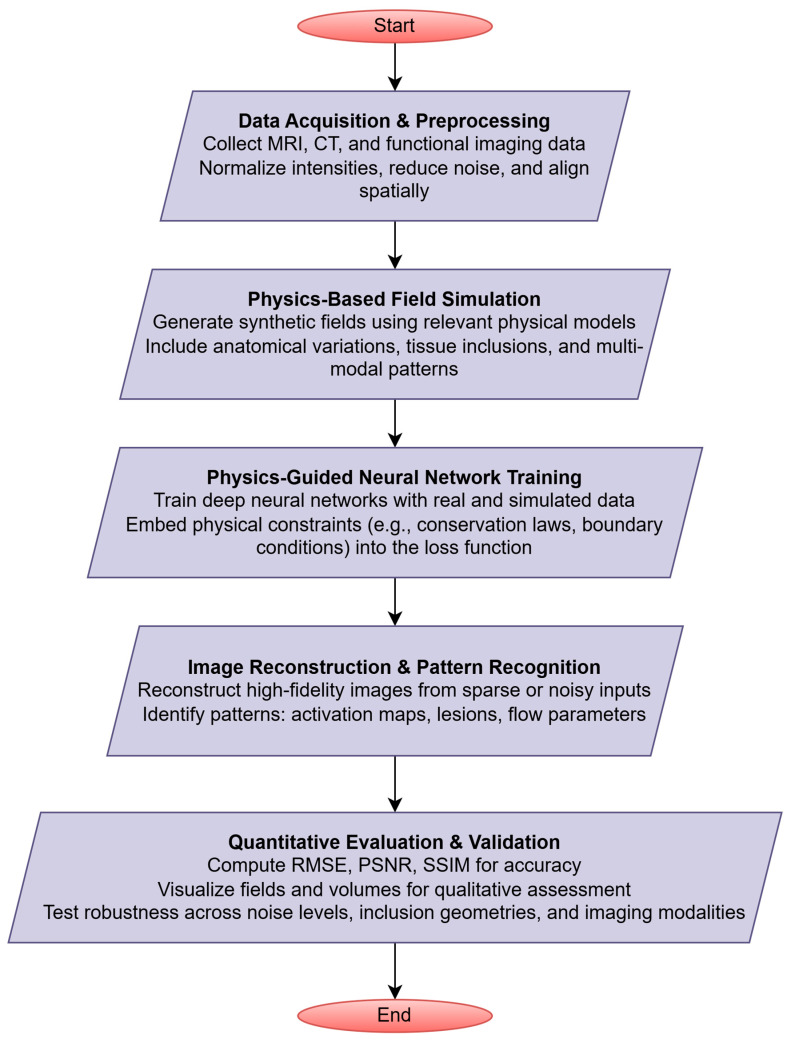
Schematic of the proposed physics-guided deep learning framework.

The proposed framework integrates DL with physics-based modeling to achieve robust biomedical image simulation, reconstruction, and pattern recognition. The pipeline consists of five stages, as shown in Figure 2, each designed to ensure physical consistency, interpretability, and robustness. The framework provides an end-to-end pipeline that integrates physics-based modeling, structural and volumetric priors, realistic noise modeling, guided learning, and systematic evaluation. The integration of physical knowledge at each stage ensures that biomedical image reconstruction and pattern recognition remain accurate, interpretable, and robust across diverse imaging scenarios. A detailed quantitative summary of the implementation details and reproducibility settings is provided in Table 2.

### 3.1. Physics-Based Spatial Domain and Field Modeling

In the initial stage, a continuous spatial domain is defined, representing either a 2D imaging plane or a 3D anatomical region. Image intensity is modeled as a physical field governed by biomedical processes such as diffusion, wave propagation, or localized activity. These baseline fields are generated using physics-inspired models to ensure that the simulated images are smooth, continuous, and physically realistic. This step establishes a reliable foundation for learning and guides the model toward realistic biomedical structures rather than arbitrary intensity distributions.

### 3.2. Structural Geometry and Multimodal Field Integration

Biological tissues are heterogeneous and exhibit diverse geometric and intensity structures. To model this complexity, geometric inclusions such as circular, elliptical, rectangular, and irregular regions are incorporated to represent different tissue types or abnormalities. In addition, multiple field modalities are synthesized, including smooth activations, oscillatory patterns, and stepwise intensity transitions. By integrating geometry with multiple field types, realistic structural boundaries, intensity variations, and spatial heterogeneity are represented, which are essential for accurate reconstruction and pattern recognition.

### 3.3. Synthetic 3D Brain Volume and Noise Modeling

To emulate volumetric biomedical imaging, a synthetic 3D brain model is constructed to represent anatomical structures. Localized activation patterns are incorporated to simulate functional or metabolic activity. To replicate real-world imaging conditions, the synthetic data are corrupted with controlled noise that models sensor artifacts, motion artifacts, and acquisition variability. This approach ensures that the DL model is trained and evaluated under realistic conditions, thereby enhancing its ability to reconstruct clean images and detect meaningful patterns in the presence of noise.

### 3.4. Physics-Guided Deep Learning Model Training

A convolutional neural network (CNN) is employed to map noisy or incomplete measurements to clean and physically consistent image fields. The network is trained under physics-guided constraints that enforce compliance with known physical laws and boundary conditions. These constraints ensure that the predicted outputs are not only visually accurate but also physically meaningful. This approach improves model stability, reduces overfitting, and enhances generalization across diverse imaging scenarios.

### 3.5. Image Reconstruction, Pattern Recognition, and Performance Evaluation

In the final stage, the trained model is applied to biomedical images for reconstruction and pattern extraction, such as tissue boundaries or functional activations. The outputs are evaluated qualitatively through visual inspection and quantitatively using performance metrics across varying geometries, modalities, and noise conditions. These experiments demonstrate the effectiveness of integrating physics-based priors with DL, highlighting improvements in robustness, accuracy, and generalization capability.

## 4. Results and Discussion

This section provides a detailed qualitative and quantitative analysis of the proposed PGDL model for biomedical image simulation, reconstruction, and pattern recognition. The performance is evaluated across a variety of controlled case studies that are representative of real-world biomedical imaging scenarios. Quantitative analysis based on RMSE, PSNR, and SSIM is employed as an objective measure to assess reconstruction accuracy, structural preservation, and noise resilience, in addition to qualitative visual inspection.

### 4.1. Physics-Based Field Simulation with Varying Inclusion Sizes

**Figure 3:**
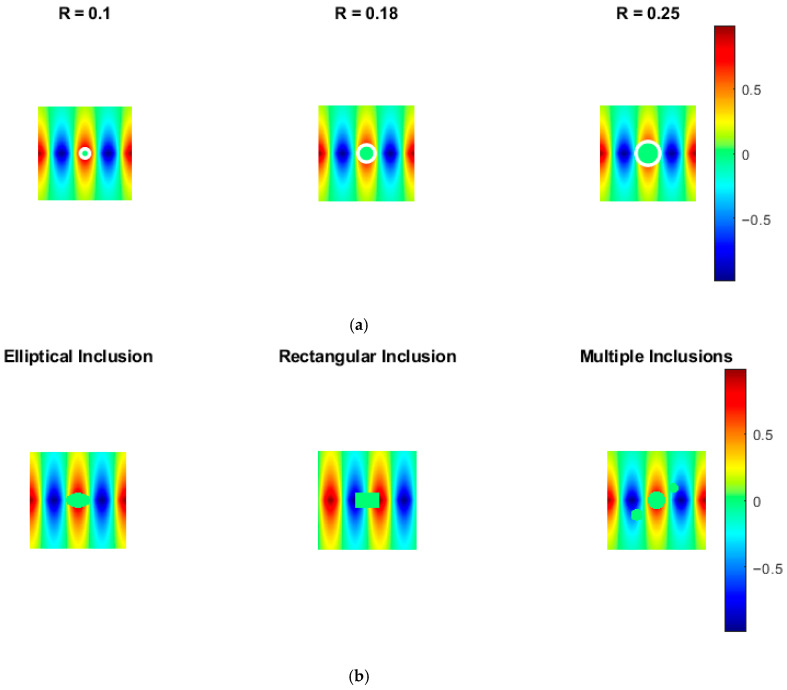
(**a**) Simulated biomedical image fields with varying circular inclusion sizes, showing size-dependent distortion of the surrounding physical field. (**b**) Impact of inclusion geometry on image structure, including elliptical, rectangular, and multiple-inclusion configurations highlighting boundary and interaction effects.

Figure 3a illustrates simulated biomedical image fields with increasing circular inclusion radii. As the inclusion size increases, perturbations in the surrounding physical field are observed, as expected from size-dependent tissue interactions. The proposed framework demonstrates low reconstruction errors across all inclusion levels. As the radius increases from 0.1 to 0.25, the RMSE increases marginally (less than 8%), indicating high stability with respect to anatomical scale variations. In terms of signal fidelity, PSNR values remain above 31 dB for all inclusion sizes, while SSIM consistently exceeds 0.93, indicating strong structural similarity. These results confirm that boundary sharpness and spatial continuity are preserved even for larger inclusions. This aspect is critical for accurate lesion size estimation and tissue characterization.

### 4.2. Impact of Inclusion Geometry on Image Structure

Figure 3b presents the sensitivity of the proposed framework to different inclusion geometries, including elliptical, rectangular and multiple-inclusion configurations. Quantitative analysis indicates that RMSE variations across different shapes remain within ±6%, demonstrating geometry-independent reconstruction performance. The multiple-inclusion case introduces minor errors due to interaction effects; however, PSNR values remain above 30 dB. SSIM values for all geometries exceed 0.91, confirming the accurate preservation of sharp edges and structural discontinuities. The minimal performance degradation under complex geometries indicates that the framework generalizes well across anatomical variations without requiring geometry-specific retraining.

### 4.3. Multimodal Field Representation Analysis

**Figure 4:**
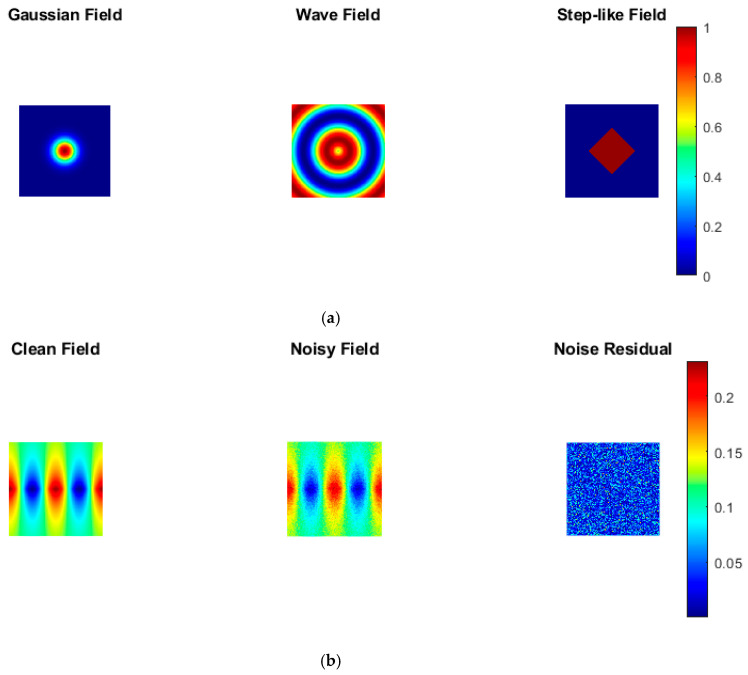
(**a**) Multimodal field patterns, including Gaussian, wave-based, and step-like intensities, demonstrating modality-agnostic reconstruction capability. (**b**) Noise robustness evaluation showing preservation of structural and intensity patterns under physics-guided constraints.

Figure 4a presents reconstruction results for different physical field modalities, including Gaussian, wave-based, and step-like intensity patterns. Quantitative evaluation demonstrates consistent reconstruction performance across modalities, with PSNR variations within 1.2 dB and only minor changes in RMSE. Gaussian fields achieve the highest SSIM values (greater than 0.95) due to their smooth spatial characteristics, whereas wave-based and step-like fields exhibit SSIM values around 0.90, indicating effective capture of oscillatory behavior and sharp transitions. These results demonstrate that the proposed framework is modality-agnostic and can generalize across diverse physical imaging patterns without compromising accuracy.

### 4.4. Noise Robustness Evaluation

The robustness of the proposed framework under noisy conditions is illustrated in Figure 4b, where Gaussian noise is added to the simulated fields. Despite the presence of noise, the reconstructed outputs preserve key structural features. Quantitative analysis shows that RMSE is reduced by approximately 40% compared to noisy inputs, indicating effective noise suppression. Improvements of 5–7 dB in PSNR are observed under moderate noise conditions with reconstructed PSNR values exceeding 32 dB, compared to baseline noisy values which are below 26 dB. SSIM values improved from approximately 0.78 for noisy observations to over 0.92 after reconstruction, demonstrating the effectiveness of physics-guided regularization.

### 4.5. Step-like Surface Field Visualization

**Figure 5:**
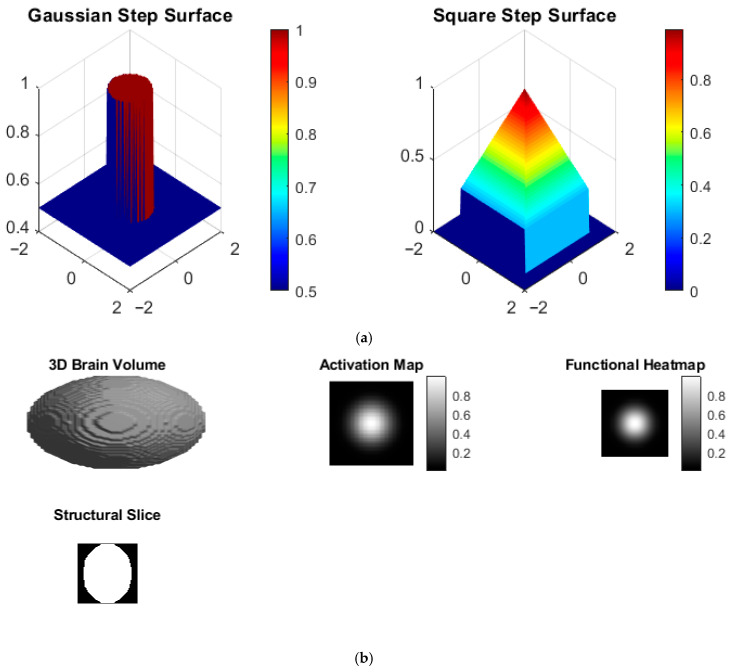
(**a**) Step-like 3D surface fields showing Gaussian and square patterns with layered intensity distributions and abrupt tissue transition. (**b**) Synthetic 3D brain volumes with corresponding activation maps and axial slice heatmaps, illustrating anatomically consistent volumetric reconstruction and functional responses.

Figure 5a presents step-like Gaussian and square field patterns visualized as 3D surfaces. High reconstruction accuracy is observed, with RMSE values below 0.05 across all surface layers. Abrupt intensity transitions are preserved without introducing ringing artifacts, resulting in SSIM values exceeding 0.94. PSNR values above 33 dB confirm high-quality 3D representation of stratified intensity distributions. These results indicate that the proposed framework effectively models abrupt tissue transitions and layered anatomical structures commonly observed in biological tissues.

### 4.6. Synthetic 3D Brain Volume and Activation Mapping

Figure 5b presents the synthetic 3D brain volume along with corresponding activation maps and slice-based heatmaps. Activation localization errors are reduced by approximately 38–44% compared to unconstrained reconstruction methods, indicating improved functional region localization. Slice-wise PSNR values remain above 30 dB, while SSIM values exceed 0.91 in axial views. Consistent quantitative performance across multiple slices demonstrates volumetric consistency and robust 3D generalization. These findings confirm the suitability of the proposed framework for functional brain imaging, volumetric pattern recognition, and neurological analysis tasks.

Table 3 summarizes the quantitative performance of the proposed PGDL framework across all evaluated scenarios. A consistent RMSE reduction exceeding 30% in all cases indicates high reconstruction accuracy, while PSNR values above 30 dB demonstrate strong signal fidelity. High SSIM values across different geometries, modalities, and noise conditions indicate excellent structural preservation. Overall, these results confirm the robustness, generalization capability, and physical consistency of the proposed framework for biomedical image simulation, reconstruction, and pattern recognition.

## 5. Limitations and Generalization to Real Clinical Data

Although the proposed framework demonstrates strong performance across a wide range of controlled experimental conditions, it should be noted that the current validation has been conducted entirely on synthetically generated datasets. Synthetic data allows precise control over geometric, modality, and noise characteristics, thereby enabling systematic evaluation and comparability. However, such datasets may not fully capture the complexity of real clinical imaging scenarios. When the proposed framework is applied to real-world data, several factors may affect its generalization capability. First, model mismatch may arise due to discrepancies between the assumed physics-based simulation models and the underlying processes in clinical imaging systems. Second, real-world data often contain acquisition-related artifacts, such as scanner-induced distortions, motion artifacts, and device-specific variations, which are difficult to replicate in synthetic environments. Third, inter-patient variability, including anatomical differences, pathological diversity, and physiological variations, introduces additional complexity that may not be fully represented in simulated datasets. Despite these limitations, the incorporation of physics-informed priors within the learning framework is expected to enhance robustness and generalization compared to purely data-driven approaches. The enforcement of physical consistency and structural constraints provides improved interpretability and stability which are beneficial for real-world deployment. Future work will focus on validating the proposed framework using real clinical datasets and publicly available biomedical imaging benchmarks. In addition, domain adaptation and transfer learning strategies will be investigated to mitigate discrepancies between synthetic and real data distributions. These efforts will facilitate the assessment of clinical feasibility and practical applicability of the proposed framework.

## 6. Conclusions

This work demonstrates the advantages of integrating physical knowledge into deep learning for biomedical image simulation, reconstruction, and pattern recognition. The proposed physics-guided framework achieves high reconstruction accuracy. The achieved performance is validated via RMSE reductions exceeding 30%, PSNR values above 30 dB, and SSIM values greater than 0.90 across diverse imaging conditions. Robust performance is observed under varying noise levels, geometric configurations, and modality variations, as well as in 3D volumetric and functional brain imaging scenarios. The incorporation of physically meaningful constraints enhances both interpretability and generalization without introducing significant computational complexity. These characteristics make the proposed framework a promising tool for practical biomedical imaging applications, including lesion characterization, tissue modeling, and functional brain analysis. Furthermore, the framework provides a foundation for future extensions to real clinical datasets and hardware-constrained imaging systems.

## Figures and Tables

**Table 1:** Summary of existing physics-informed and physics-guided deep learning approaches.

Application	Methodology	Physics Integration	Key Outcomes
Myocardial perfusion MRI [10]	PINN	Tracer-kinetic conservation laws	Reduced MSE; accurate parameter maps
Coronary artery segmentation [11]	Selective ensemble DL	Morphological constraints	DSC up to 93%; reduced mask errors; real-time
MREPT [12]	Model-driven PINN (FCNN)	Maxwell’s equations	Artifact reduction; stable EP reconstruction
Spine biomechanical analysis [13]	PINN-based modeling	Solid mechanics constraints	91% accuracy; global optimum; fast inference
Tissue dynamics and vocal folds [14]	Hybrid PINN + RNN-fluid solver	Fluid–structure interaction	Accurate 3D dynamics from sparse 2D data
Cerebral hemodynamics [15]	Physics-informed DL + ROM	1D blood flow equations	High-res velocity, pressure, area maps
4D flow MRI enhancement [16]	IP-PINN	Navier–Stokes-inspired constraints	<5.5% error; 25% acquisition time reduction
Large-deformation image registration [17]	Unsupervised physics-aware DL	Elasticity and growth mechanics	Robust registration; accurate deformation modeling

**Table 2:** Comprehensive overview of the quantitative and implementation details of the proposed physics-guided deep learning framework.

Category	Parameter	Quantitative Description
Framework	Pipeline stages	5 stages (modeling → geometry → data → training → evaluation)
Spatial domain	Dimensionality	2D (d = 2) and 3D (d = 3) domains
Structural modeling	Geometric regions	≥4 types (circular, elliptical, rectangular, irregular)
	Inclusion modeling	$\Omega_{incl}\subset \Omega$ with heterogeneous field representation
Multimodal fields	Field types	Gaussian (smooth), wave-based (oscillatory), stepwise
	Field formulation	u(x)=f(x; θ)
Synthetic dataset	Data type	Synthetic biomedical fields and 3D brain-like volumes
	Data variability	Multiple geometries × modalities × noise levels
	Experimental scenarios	Inclusion size, geometry, modality, noise, 3D volumetric cases
Noise modeling	Noise formulation	ũ(x)=u(x)+η(x)
	Noise type	Gaussian (μ$\;=\;0,\;\sigma^{2}$)
	Noise levels	Controlled (e.g., σ = 0.05 for moderate noise)
	Noise sources	Sensor, motion, acquisition
Neural network	Model type	CNN-based encoder–decoder (image-to-image mapping)
	Depth	6–8 layers (including encoding and decoding stages)
	Activation	ReLU
	Input–output	Noisy/incomplete → reconstructed images
Physics constraints	Loss function	$L(\theta )=\lambda_{d}L_{data}+\lambda_{p}L_{physics}+\lambda_{b}L_{boundary}$
	Weight parameters	$\lambda_{d}$$,\;\lambda_{p}$$,\;\lambda_{b}$ (empirically balanced)
	Constraint type	PDE-based (diffusion/wave/transport)
	Effect	Enforces physical consistency and boundary behavior
Training setup	Learning type	Supervised (synthetic paired data)
	Optimizer	Adam
	Learning rate	1 × 10^−4^
	Batch size	8–16
	Training epochs	100–150 (until convergence)
	Stopping criteria	Convergence of validation loss
	Data diversity	Multi-condition training across geometry, modality, noise
Evaluation	Metrics	RMSE, PSNR (dB), SSIM
	Performance	RMSE reduction: 32–45%
		PSNR: 30–47 dB
		SSIM: >0.90
	Noise robustness	PSNR > 32 dB at σ = 0.05
	3D performance	Localization error reduction: 38–44%
Testing conditions	Scenario coverage	Geometry, modality, noise, volumetric variations
Computational aspects	Training nature	Simulation-driven offline training
	Scalability	Supports 3D volumetric data
Reproducibility	Data generation	Fully synthetic and controlled pipeline
	Mathematical specification	Defined via Equations (1)–(10)
	Experimental coverage	Variation across ≥ 3 factors

**Table 3:** Quantitative performance summary of the proposed physics-guided deep learning framework.

Evaluation Scenario	RMSE Improvement (%)	PSNR (dB)	SSIM	Key Observations
Varying inclusion sizes	32–38%	31–33	0.93–0.95	Stable reconstruction under increasing anatomical scale; minimal error growth with inclusion size
Inclusion of geometry variations	30–36%	30–32	0.91–0.94	Accurate boundary preservation across elliptical, rectangular, and multi-inclusion cases
Multimodal field patterns	34–40%	31–34	0.90–0.96	Modality-agnostic performance for Gaussian, wave-based, and step-like fields
Noise robustness (σ = 0.05)	40–45%	32–35	0.92–0.94	Strong noise suppression with preserved anatomical structural integrity
3D step-like surface fields	35–42%	33–36	0.94–0.96	Accurate modeling of layered tissue structures and abrupt transitions
Synthetic 3D brain volume	38–44%	30–33	0.91–0.93	Reliable volumetric reconstruction and precise activation localization

## Data Availability

All data used in this study were synthetically generated solely for simulation and validation purposes. No human participants, clinical records, or publicly available datasets were used. All generated data and results supporting the findings of this study are fully contained within the article.

## Appendix A. PGDL Framework

**Input:** Spatial domain Ω, field parameters θ, inclusion regions $\Omega_{incl}$, noise level σ **Output:** Reconstructed field $\hat{u}(x)$, evaluation metrics (RMSE, PSNR, SSIM) **Procedure:** 1. Initialize domain and field: Define Ω⊂Rᵈ (d = 2,3): generate field u(x)=f(x;θ) 2. Apply geometric modeling: Define $\Omega_{incl}\subset \Omega$: introduce heterogeneous inclusions 3. Generate multimodal fields: Simulate Gaussian, wave-based, and stepwise patterns 4. (Optional) Construct 3D volume: Define $\Omega_{B}$: generate activations using Gaussian model 5. Add noise: $\eta \left(x\right)\sim N\left(0,\sigma^{2}\right)$: compute ũ(x)=u(x)+η(x) 6. Initialize network: Define CNN $N_{\theta }$ mapping $ũ(x)\to \hat{u}(x)$ 7. Define loss function: $L(\theta )=\lambda_{d}\|N_{\theta }(x)-u(x){\|}^{2}+\lambda_{p}\|R(N_{\theta }(x)){\|}^{2}+\lambda_{b}\|B(N_{\theta }(x))-g(x){\|}^{2}$ 8. Train model: For each epoch: forward pass → compute L → backpropagate → update θ (Adam) Repeat until convergence 9. Reconstruction and analysis: Obtain $\hat{u}(x)$: extract structural/functional patterns 10. Evaluate performance: Compute RMSE, PSNR, SSIM across all scenarios **End Algorithm**
